# Genetic manipulation allows in vivo tracking of the life cycle of the son‐killer symbiont, *Arsenophonus nasoniae*, and reveals patterns of host invasion, tropism and pathology

**DOI:** 10.1111/1462-2920.14724

**Published:** 2019-07-11

**Authors:** Pol Nadal‐Jimenez, Joanne S. Griffin, Lianne Davies, Crystal L. Frost, Marco Marcello, Gregory D. D. Hurst

**Affiliations:** ^1^ Institute for Integrative Biology University of Liverpool Liverpool UK

## Abstract

Maternally heritable symbionts are common in arthropods and represent important partners and antagonists. A major impediment to understanding the mechanistic basis of these symbioses has been lack of genetic manipulation tools, for instance, those enabling transgenic GFP expression systems for in vivo visualization. Here, we transform the ‘son‐killer’ reproductive parasite *Arsenophonus nasoniae* that infects the parasitic wasp *Nasonia vitripennis* with the plasmid pOM1‐*gfp*, re‐introduce this strain to *N. vitripennis* and then used this system to track symbiont life history in vivo. These data revealed transfer of the symbiont into the fly pupa by *N. vitripennis* during oviposition and *N. vitripennis* larvae developing infection over time through feeding. A strong tropism of *A. nasoniae* to the *N. vitripennis* ovipositor developed during wasp pupation, which aids onward transmission. The symbiont was also visualized in diapause larvae. Occasional necrotic diapause larvae were observed which displayed intense systemic infection alongside widespread melanotic nodules indicative of an active but failed immune response. Our results provide the foundation for the study of this symbiosis through in vivo tracking of the fate of symbionts through host development, which is rarely achieved in heritable microbe/insect interactions.

## Introduction

Current estimates indicate that heritable bacterial symbionts are present in over 50% of all arthropod species, with presence defined as being carried by more than 0.1% of individuals in the species (Weinert *et al*., 2015). These symbionts, which in the majority of cases pass from a female host to her offspring, have been shown to critically affect the biology of their hosts in multiple ways: providing essential nutrients (Shigenobu *et al*., 2000; Pais *et al*., 2008; Hosokawa *et al*., 2010), protecting against predators, parasites and pathogens (Oliver *et al*., 2003; Teixeira *et al*., 2008; Osborne *et al*., 2009; Nakabachi *et al*., 2013), or inducing reproductive abnormalities (see (Hurst and Frost, 2015) for review). These impacts on host individuals drive symbiont infection into host populations, and modify the biology, ecology and evolution of their host species. The phenotypes also enable endosymbionts to be used as a ‘Trojan horse’ against vector‐borne diseases or to fight against insect pests (De Vooght *et al*., 2018; O'Neill *et al*., 2018).

Investigating the interaction between heritable microbes and their insect hosts is commonly an onerous activity. Most insect endosymbionts have undergone a process of genome minimization resulting in their inability to grow outside their host in cell‐free culture (Moran *et al*., 2008). Thus, standard genetic techniques such as gene knock outs, and transformation with plasmids that express GFP or other properties, have not been achieved. The lack of gene knock outs makes loss of function analysis dependent on naturally occurring mutations (Oliver *et al*., 2009; Harumoto and Lemaitre, 2018). Gain‐of‐function assays are restricted to laborious transgenic expression in the host that have restricted research to well‐established model insects (Beckmann *et al*., 2017; LePage *et al*., 2017; Harumoto and Lemaitre, 2018). The inability to express fluorophores confines visualization of symbionts to fixed material, using antibody or FISH‐based techniques.

Five heritable microbial symbionts, the Mollicute *Spiroplasma poulsonii*, the alphaproteobacteria *Asaia*, and the gammaproteobacteria *Arsenophonus nasoniae*, *Hamiltonella defensa* and *Sodalis glossinidius*, present a contrast in that they can be grown in cell‐free culture (Werren *et al*., 1986; Dale and Maudlin, 1999; Matthew *et al*., 2005; Favia *et al*., 2007; Brandt *et al*., 2017; Masson *et al*., 2018). These bacteria present an opportunity to study diverse microbe‐insect symbioses, as these symbionts are themselves reproductive parasites [male‐killing in *S. poulsonii*, *A. nasoniae*: (Werren *et al*., 1986; Montenegro *et al*., 2005)], protective symbionts [*S. poulsonii*, *H. defensa*: (Oliver *et al*., 2003; Xie *et al*., 2014)] and secondary symbionts affecting vector competence [*S. glossinidius*: (Dale and Welburn, 2001)]. Further, they are related to a range of microbes with diverse and important symbiotic interactions with their host (e.g., defensive, anabolic, reproductive parasitic). To date, genetic manipulation has been limited to *S. glossinidius* and *Asaia*, In *Sodalis*, gain‐of‐function assays have been achieved through expression on plasmids (Weiss *et al*., 2008), and loss‐of‐function knockout of focal genes completed using directed and random mutagenesis (Dale *et al*., 2001; Pontes and Dale, 2011; Pontes *et al*., 2011; Hrusa *et al*., 2015). These modified strains can be readily re‐established in their host in vivo to analyse function (De Vooght *et al*., 2018). In *Asaia*, transformation with plasmids expressing GFP has been completed, permitting strains to be tracked in vivo without fixation (Favia *et al*., 2007; Damiani *et al*., 2008; Crotti *et al*., 2009).

Application of a greater range of genetic manipulations to a wider array of symbionts would enable in vivo tracking of symbiont–host interactions, and forward genetic analysis of symbiont fate and phenotype. These techniques would then enable research into the genetic systems underlying the development and maintenance of symbiotic lifestyles, and the mechanisms underlying symbiont impact on their hosts. The systems developed would additionally potentiate paratransgenic application (Gilbert *et al*., 2016).

In this article, we establish the plasmid pOM1‐*gfp* (Basset *et al*., 2003) in *A. nasoniae* and use this to visualize the symbiotic process in vivo. *Arsenophonus nasoniae* is the son‐killer symbiont of the jewel wasp *Nasonia vitripennis* (Gherna *et al*., 1991). The microbe belongs to the male‐killer family of symbionts, and the death of male offspring is achieved through an as yet unidentified diffusible toxin that alters the behaviour of the host's maternal centrosome (Ferree *et al*., 2008). Previous work tracked the development of the symbiosis in fixed material through light microscopy alongside TEM images (Huger *et al*., 1985). The symbiont shows maternal inheritance without invasion of eggs and is thought to infect larvae following feeding on the fly pupa, that is contaminated during wasp oviposition (Huger *et al*., 1985). This pattern of transmission also enables the horizontal spread of the bacterium both within species (when two *N. vitripennis* females utilize the same fly host) and between species (when different wasp species utilize the same fly host) (Skinner, 1985; Duron *et al*., 2010; Parratt *et al*., 2016). Infection is extracellular in larvae and adults, which makes the symbiosis distinct from many heritable microbes, in which the microbes are obligately intracellular.

We transformed *A. nasoniae* with pOM1‐*gfp*, verified the stability of the plasmid during in vivo passage without selection, and then present data tracking infection progression in vivo from the point at which the wasp oviposits into a pupa to the time at which the adult wasp emerges.

## Results and discussion

### *pOM1‐*gfp *is maintained in* A. nasoniae *in vivo without selection*

*Arsenophonus nasoniae* carrying pOM1*‐gfp* were successfully established in vitro, evidenced by bright‐green fluorescence of colonies on plates compared with controls (Supporting Information Fig. S1), and the strain created is henceforth referred as *A. nasoniae* Fin'13 pOM1‐*gfp* and hereafter abbreviated as *An*‐GFP.

In vitro growth of *An*‐GFP and the WT progenitor was comparable, indicating the cost of plasmid carriage to *A. nasoniae* was low (Supporting Information Fig. S2).

**Figure 1:**
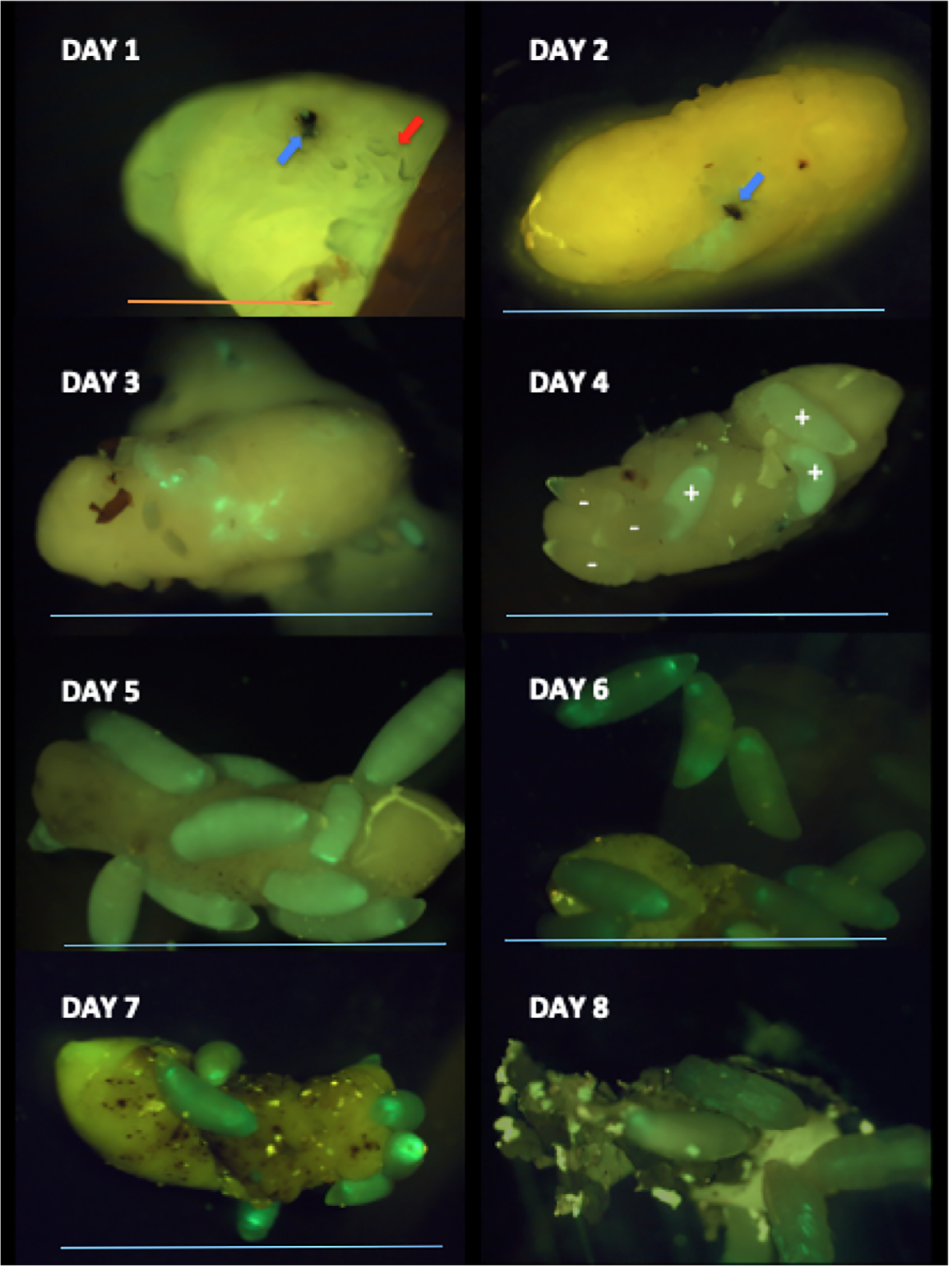
Progression of An‐GFP infection of fly pupa cadaver and *Nasonia vitripennis* larvae following oviposition by *An*‐GFP infected mothers. Time series (from days 1 to 8) is represented as one plate per day post‐oviposition. At day 1, the site of oviposition is marked by the melanotic damage mark (blue arrow) and *N. vitripennis* eggs proximate to this (red arrow), but fluorescence indicative of *An*‐GFP growth is no more than a pinprick. At day 2, growth of *An*‐GFP on the fly pupal cadaver surface can be observed (green fluorescent patch) and the eggs have hatched into wasp larvae. By day three, larvae can be found feeding on the surface of the pupae on which *An*‐GFP colonies are evident, and a minority of larvae have established *Arsenophonus nasoniae* infection. At day 4, there is a mix of *An*‐GFP infected (+) and *An*‐GFP uninfected (−) within a single fly pupa. *An*‐GFP colonies on the surface are now not apparent and have likely been consumed. The majority of larvae become infected by day 7–8, at which point infections commonly have a strong oral tropism. Scale: blue bar = 10 mm long, orange bar = 3 mm. [Color figure can be viewed at wileyonlinelibrary.com]

*An*‐GFP were re‐introduced to *N. vitripennis* through injecting fly pupae with 0.5–1 μl of liquid culture, and then allowing *N. vitripennis* to oviposit on the fly pupa following methods developed by Werren (1986). The presence of *An*‐GFP in *N. vitripennis* pupae was established by ‘cracking’ the fly pupa 10 days after *N. vitripennis* oviposition and examining under epifluorescence. *An*‐GFP infection was indicated by bright‐green fluorescence of *N. vitripennis* pupae (see Fig. 1 for example pictures), which contrasted with dull‐yellow autofluorescence of wasp pupae lacking *A. nasoniae* (see Supporting Information Fig. S3 for example pictures).

Plasmids may segregate and be lost in the absence of selection or plasmid maintenance systems. We determined the stability of pOM1‐*gfp* in *A. nasoniae* by tracking the persistence of the strain over several generations of passage in *N. vitripennis*. To this end, we introduced *An*‐GFP into *N. vitripennis* in two replicates, passaged it through 10 *N. vitripennis* generations without selection, and examined wasp pupae at generation G10 for GFP presence through epifluorescence. Stability was confirmed in both cases, and these lines have now retained infection through over 25 host generations.

The male‐killing efficiency of the symbiont was assessed in terms of (i) the sex ratio produced by *An*‐GFP vs uninfected mated females ovipositing singly and (ii) the number of sons produced by *An*‐GFP vs uninfected virgin females (note virgin females produce only sons; the relative number of sons then estimates male‐killing efficiency). In the mated female assay, *An*‐GFP infected females produced 2.2% sons (*n* = 228) compared with 14.4% sons produced by uninfected females (*n* = 449). In the virgin female assay, *An*‐GFP infected females produced on average 8.38 surviving sons (s.e. = 0.46), compared with 23 sons (s.e. = 1.35) produced by uninfected females. Male‐killing efficiency of *An*‐GFP was estimated at 86% in the mated female assay and at 63.5% in the virgin female assay.

### Arsenophonus nasoniae *is exclusively inherited through females*

As for the majority of other endosymbionts, *A. nasoniae* has been proposed to be exclusively inherited through females, leaving males as a dead end for the endosymbiont. For *A. nasoniae* these conclusions were reached using defined crosses and tracking the son‐killer phenotype (rather than the symbiont itself; Skinner, 1985).

*An*‐GFP carrying *N. vitripennis* were used to verify these results through tracking the symbiont directly. Fifteen male *N. vitripennis* carrying *An*‐GFP were mated to virgin females that did not harbour infection, and 10‐day old G1 wasp pupae retrieved and visualized. In all cases, all *N. vitripennis* pupae resulting from these crosses were negative for GFP. Individuals of G2 and G3 generations of these crosses were likewise negative, indicating that *A. nasoniae* is passed through oviposition alone, and is neither paternally nor sexually transmitted.

### Arsenophonus nasoniae *infection development tracked using GFP*

We utilized *An*‐GFP to examine the symbiosis between *A. nasoniae* and *N. vitripennis* through wasp development. We allowed sets of five *N. vitripennis* mated females carrying *An*‐GFP established above to oviposit for 24 h on five fly pupae, and then tracked the progression of infection both on the fly pupa and during *N. vitripennis* development. Oviposition events were replicated, such that data were obtained for 3–15 events per day over 13 days of *N. vitripennis* development. In total, 111 fly pupae were parasitized by wasps from *An*‐GFP‐positive lines, and *An*‐GFP infection transfer to the fly pupa was evident in 103 of these (as scored as either the fly pupa or *N. vitripennis* G1 progeny being infected). These data were compared with development of *N. vitripennis* without *A. nasoniae* that were established to determine patterns of autofluorescence.

*Nasonia vitripennis* life history begins when a female stings a fly pupa, injecting venom, and then oviposits 5–50 eggs into it. The sites at which the fly pupae were stung by *N. vitripennis* were easily recognized by the presence of dark‐melanized tissue around this area (Fig. 1, days 1–3). Previous experiments and light microscopy indicated *A. nasoniae* was not transmitted inside eggs (Huger *et al*., 1985). Compatible with this, a pinprick size *An*‐GFP infection could be observed at the zone where wasp females injected venom to arrest the development of the fly pupa. Eggs were observed near, but not at the injection site, and *An*‐GFP was not observed in or around the eggs. On the second day, the intensity of GFP visible at the stinging site was greater when compared with that of the previous day indicating that *An*‐GFP had replicated in this period. Expansion of *An*‐GFP infection on the fly pupal surface was not observed beyond day 3 in our time series, and *An*‐GFP was never observed to spread over the surface of the fly pupae beyond the point of initial inoculation. Control fly pupae (stung by wasps without *A. nasoniae*) showed a dull‐yellow pattern of autofluorescence over the entire fly pupal surface (Supporting Information Fig. S3), which was readily distinguished from the bright‐green area observed in the presence of *An*‐GFP.

We conclude that *An*‐GFP growth is restricted to the venom/calyx fluid at the immediate area in which the wasp stings the host, and we hypothesize the microbe may be dependent on the calyx fluid to support growth. The very small initial size of the *An*‐GFP colony indicates that *A. nasoniae* undergoes a bottleneck in terms of numbers transferred during oviposition.

Infection of *N. vitripennis* larvae by *An*‐GFP occurred progressively over time through feeding on the infected pupa (Fig. 1, Supporting Information Fig. S4). Initially hatched larvae (day 2) showed no evidence of *An*‐GFP infection, and we were unable to differentiate these larvae from negative controls in terms of GFP fluorescence. Wasp larvae feed through roaming over the fly pupal surface, and did so without apparent attraction/avoidance of the area of the pupa infected with *An*‐GFP. Infection was acquired progressively over time. On day 3, 15% of wasp larvae showed signs of *An*‐GFP infection, and at this time, infected and uninfected wasp larvae were observed within the same brood. The fraction of infected wasp larvae increased to over 95% by day 8, the time at which *N. vitripennis* maggots begin to enter pupation. Controls with *N. vitripennis* lacking *An*‐GFP show a dull‐yellow autofluorescent signal in the wasp larvae (Supporting Information Fig. S3), clearly distinct from the green signal observed in the presence of *An*‐GFP.

The pattern of infection of *An*‐GFP within *N. vitripennis* larvae was variable. Initially, infection was concentrated around the mouth area, but was also visible along the digestive tract. In a minority of occasions, the infection became systemic and intense with the larvae glowing bright green along their entire length. The fraction of all infections that were systemic was heterogeneous amongst days post‐oviposition (*χ* ^2^ = 29.3, 3 d.f., *p* < 0.001), with systemic infections being more common earlier in development (Supporting Information Fig. S3).

**Figure 2:**
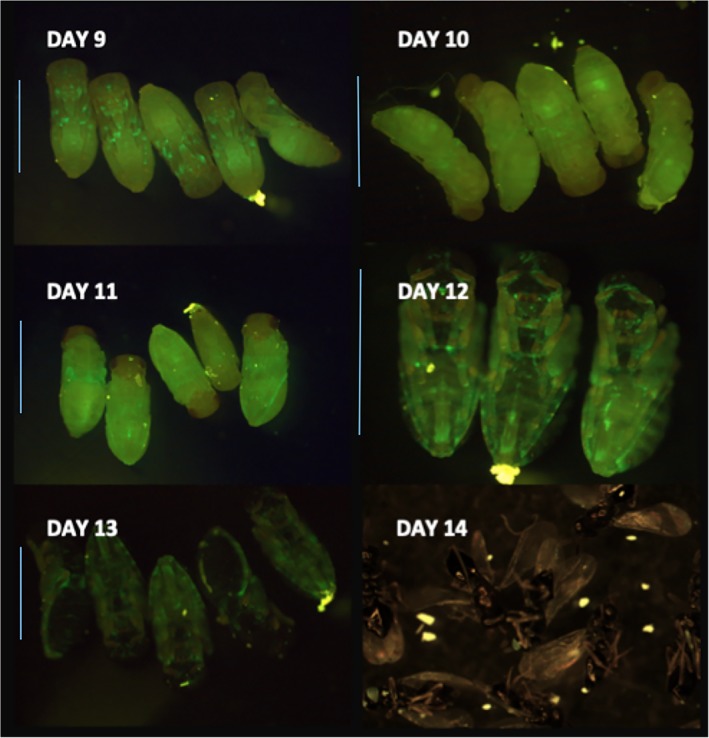
Progression of *An*‐GFP infection of *Nasonia vitripennis* pupae, 9–13 days post‐oviposition. Initially, spots of infection are observed in mouth, legs and abdomen. Formation of the *N. vitripennis* ovipositor is observed at days 11–13, and infection of *An*‐GFP of this apparatus is apparent in day 12 pupae. Scale: line represents 2 mm. [Color figure can be viewed at wileyonlinelibrary.com]

**Figure 3:**
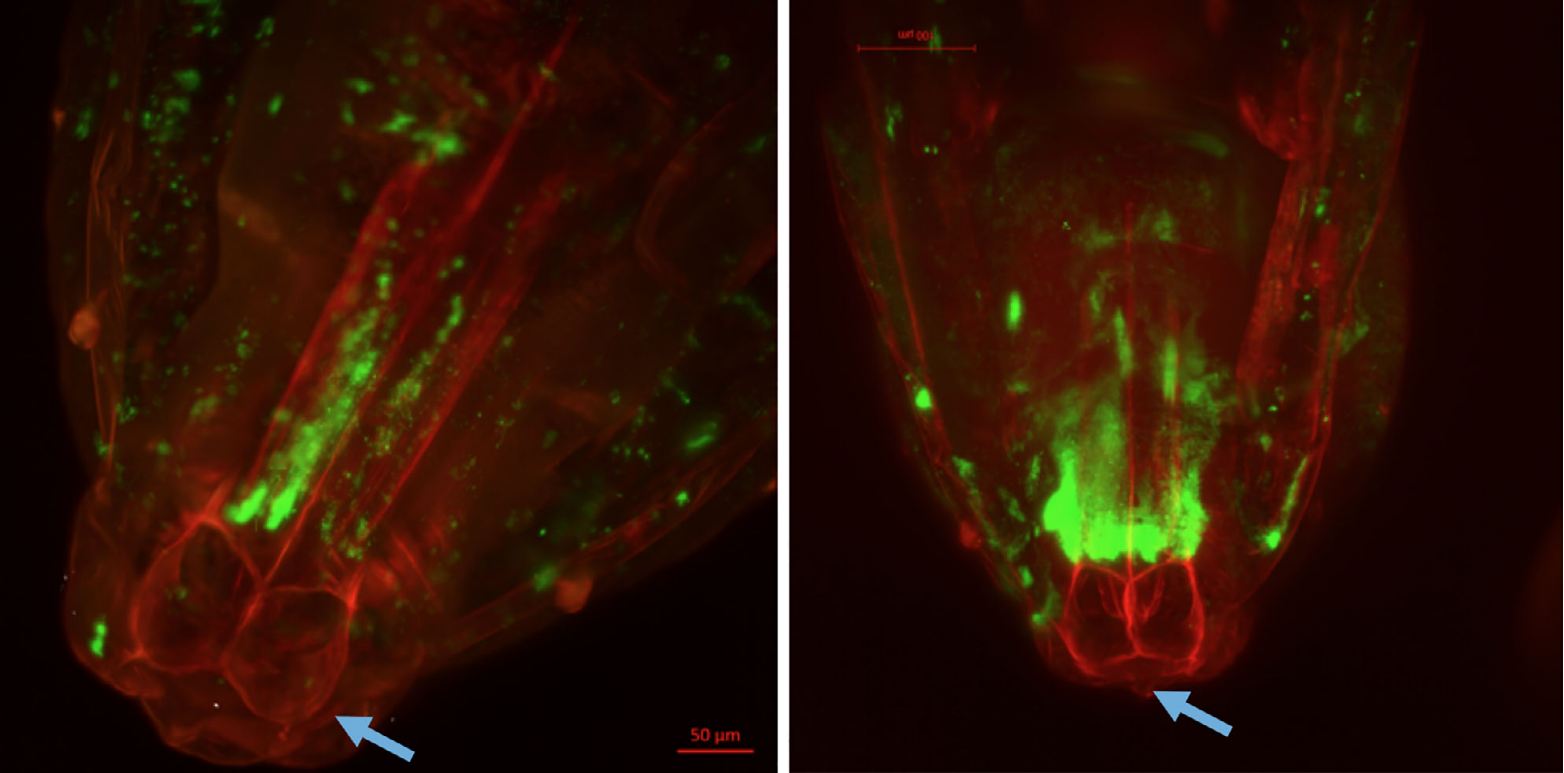
Light‐sheet microscopy images of the abdomen of two female *Nasonia vitripennis* pupae infected with *An*‐GFP at day 11. The twin tubes of the forming oviposition apparatus (arrowed) can be observed to carry high intensity of *An*‐GFP fluorescence compared with neighbouring tissues, indicating a strong tropism for this apparatus that is required for vertical transmission of the symbiont. Scale: red bar = 50 μm. [Color figure can be viewed at wileyonlinelibrary.com]

*Nasonia vitripennis* larvae developing at 25°C enter pupation from day 8–9. *An*‐GFP establishment was observed in 97.8% of wasp pupae (*N* = 505), which is consistent with the vertical transmission rate calculated previously from the inheritance pattern of the female‐biased ‘son‐killer’ phenotype (Skinner, 1985). The pupal phase of insect development is associated with progressive differentiation of adult structures within the wasp puparium, and also changes in cuticle melanism from translucent white at entry to pupation, to a melanized abdomen, to a fully melanized body (which prevents direct visualization of the endosymbiont). *An*‐GFP can be observed unequally distributed throughout the body of *N. vitripennis* pupae (Fig. 2). Importantly, *An*‐GFP shows a pronounced tropism to the oviduct and around the sting apparatus as this structure develops at day 11. We used light‐sheet microscopy to localize *An*‐GFP in 11‐day old *N. vitripennis* pupae more precisely. The results clearly reveal that *An*‐GFP is concentrated in the ovipositor tubes (Fig. 3).

**Figure 4:**
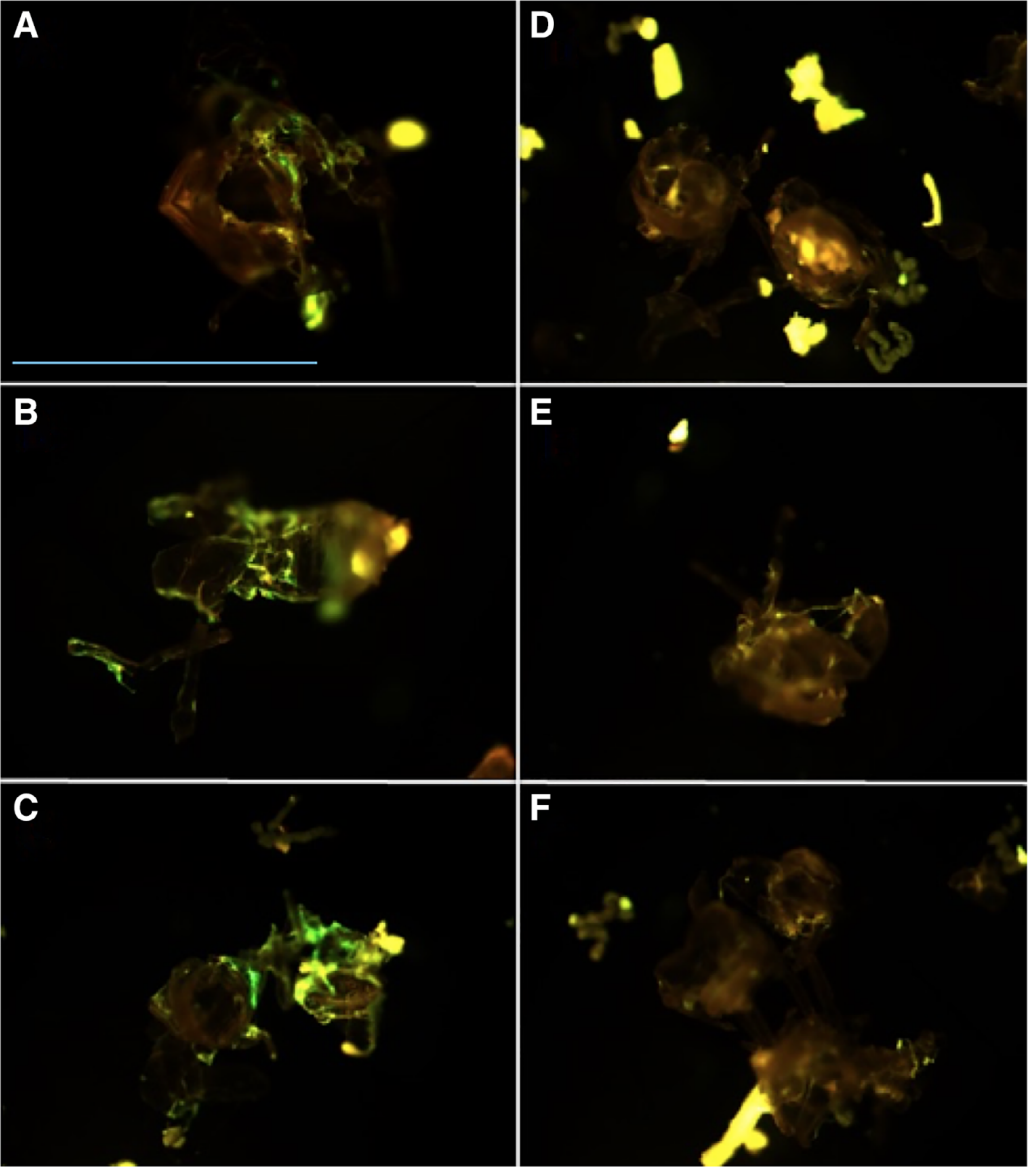
Pupal exuviae (exoskeleton cast off following ecdysis) of *Nasonia vitripennis* infected with *An*‐GFP (left‐hand side, A–C) and uninfected (right‐hand side, D–F) visualized under epifluorescence. Scale bar (blue, block A) = 2 mm. [Color figure can be viewed at wileyonlinelibrary.com]

Adult wasps emerge from their pupal case between days 13 and 15. A close examination of the exuviae (discarded pupal cases) reveals clusters of *An*‐GFP on the interior of the pupal cases (Fig. 4). Growth on the inside surfaces of the pupal case can also be clearly observed under Light‐Sheet microscopy where infection foci can be observed both within the developing adult wasp, and outside of the wasp inside the pupal case (Fig. 3). These data indicate that *A. nasoniae* adheres to, and grows externally to, the developing adult wasp within the puparium. It is possible that growth here is enabled by lack of immune activity, and that the microbe has an affinity for chitinous structures. This observation is consistent with a wide array of chitin binding proteins in the *A. nasoniae* genome (Darby *et al*., 2010).

The adult *N. vitripennis* body is highly melanized, and thus the pattern of *A. nasoniae* infection cannot be visualized in vivo. However, a single occasion when the ovipositor of an infected female became detached at the point of oviposition (and remained attached to the fly pupa) presented an opportunity to visualize *A. nasoniae* infection in this adult structure (Supporting Information Fig. S6C and D). The space within the ovipositor showed a strong positive *An*‐GFP signal, consistent with the onward transmission of *A. nasoniae* occurring extracellularly during stinging of the fly pupa. We removed the ovipositor from *An*‐GFP infected *N. vitripennis* and compared these to the same structure from uninfected females. *An*‐GFP colonization of this tissue was again indicated (Supporting Information Fig. S6A vs. B).

### Arsenophonus nasoniae *infection during* N. vitripennis *diapause*

*Nasonia vitripennis*, like many insects, enters diapause to survive winter stress (Paolucci *et al*., 2013). We sought to investigate whether *A. nasoniae* survives during *N. vitripennis* diapause as well as the potential beneficial or detrimental consequences of symbiotic infection during diapause. In this species, diapause arrest occurs as L3 larvae within fly pupae, and we induced entry into diapause through placing individual maternal females into 8:16 L:D and 15°C for 10 days, then allowing them to oviposit individually, and then assessing *A. nasoniae* infection patterns in the resulting diapause arrested *N. vitripennis* larvae.

Eighty‐seven percent of larvae in infected broods (*n* = 31 broods, *n* = 900 larvae) carried *An*‐GFP infection. We found similar tropisms to the non‐diapausing larvae, with foci of infection evident in the mouthparts and gut. Some broods displayed a mix of larvae with tropisms and systemic infections and a few broods were exclusively systemically infected. In total, 137/754 diapause larvae (18.1%, CI 15.5%–21.1%) showed systemic infections, fluorescing green throughout the body.

**Figure 5:**
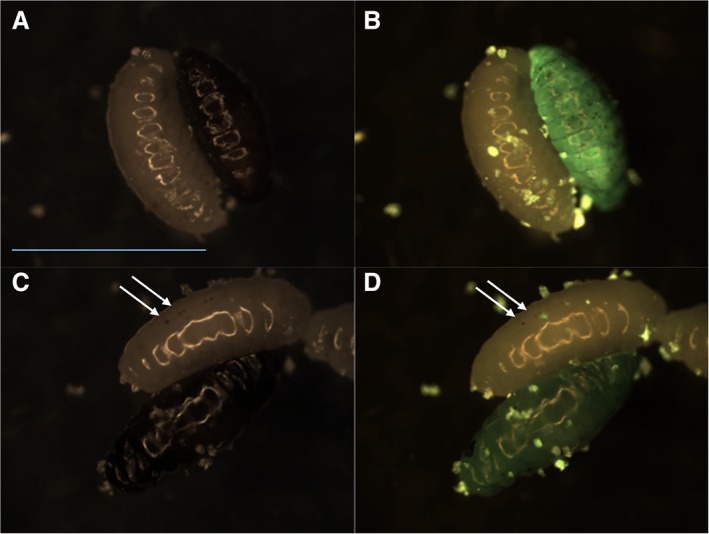
*An*‐GFP infection in necrotic (black) and live (white) diapausing *Nasonia vitripennis*. A and C – healthy/necrotic pairs in white light, B and D – epifluorescence image of same individuals. Necrotic larvae fluoresce brightly with *An*‐GFP and show melanization and thus activation of the immune system (B). Live larvae also show melanin deposits at the site of *An*‐GFP infection indicated by the arrow (C, D). Scale bar (blue, block A) = 2 mm. [Color figure can be viewed at wileyonlinelibrary.com]

Three out of the 31 *An*‐GFP diapause broods examined contained necrotic larvae, which were not observed in any of the 37 control broods where *A. nasoniae* was absent. In total, 11 necrotic larvae were observed in 754 infected larvae (1.46%, CI 0.73%–2.6%), and these glowed intensely with *An*‐GFP indicating the symbiont remained viable and had grown to higher titre than in viable wasp pupae (Fig. 5A and B). The necrotic larvae presented signs of melanized nodules throughout the body. Further to this, living diapause larvae were also observed to display melanized spots in the areas surrounding *An*‐GFP growth (Fig. 5C and D), and these melanized spots were not observed in any of the control uninfected *N. vitripennis* larvae.

From these data, we conclude *An*‐GFP interacts with the *N. vitripennis* immune system during diapause, inducing nodule formation, and that symbiont proliferation is usually maintained ‘in check’ by this. Occasionally, proliferation is observed alongside a widespread nodulation response and necrotic death of the host. The lack of necrotic death in *N. vitripennis* uninfected with *A. nasoniae* indicates that *A. nasoniae* drives these occasional deaths, which occur when *An*‐GFP is not suppressed by the nodulation response.

### *Synthesis*

Previous study describing the *A. nasoniae* – *N. vitripennis* interaction was completed through painstaking examination of fixed material and transmitted light microscopy to examine the symbiosis (Huger *et al*., 1985). The absence of infection in eggs within that study led the authors to hypothesize that vertical transmission was mechanical and occurred during the stinging/oviposition process. Our observation demonstrate this hypothesis is true, with our visualization establishing very local growth of *A. nasoniae* in the fly pupa at the point at which the wasp stings.

Oral uptake by *N. vitripennis* larvae, inferred from widespread bacterial infection of the midgut in previous studies, is confirmed in our study, with larval mouthparts being the earliest tissues showing infection. Our study details considerable heterogeneity in the timing and extent of *An*‐GFP infection within broods. Host genetic factors can be excluded as a cause of heterogeneity as a single isofemale inbred *N. vitripennis* line was used in the experiments. Experimental variation can also be ruled out, as *N. vitripennis* larvae with systemic infection and those with and without localized infection could be retrieved from the same fly pupal cadaver (e.g., Fig. 1, day 4). Rather, the roaming movement patterns of *N. vitripennis* larvae over the surface of the pupa presents a tempting causal driver for the heterogeneity in timing/extent of *A. nasoniae* invasion into larvae, and of segregational loss. Individual *N. vitripennis* larvae feeding over the surface of the fly pupa will encounter the *An*‐GFP patch sequentially over time, and some will not be exposed to infection. Stochasticity in the time of encounter with *An*‐GFP, and the amount of *An*‐GFP consumed, are likely the cause of heterogeneity in the timing and intensity of *An*‐GFP infection amongst *N. vitripennis* larvae, and provide a mechanism for segregational loss (lack of encounter of an infectious dose).

Onward transmission of *A. nasoniae* is facilitated by a profound tropism to the ovipositor that is evident during pupal development, and persists in the adult wasp. Processes for assuring vertical transmission are well recognized for transovarially transmitted microbes, and these symbionts either invade the germ line (Frydman *et al*., 2006) or cross the ovariole wall to enter developing eggs (Herren *et al*., 2013). Our case represents an example of a vertically transmitted extracellular symbiont with an adaptive mechanism for ensuring vertical transmission during oviposition. How the tropism to the ovipositor is established is unknown. In our study, the presence of *An*‐GFP growth foci in the discarded puparium case leads us to hypothesize that this pattern is a result of a growth preference of *A. nasoniae* on developing chitinous structures, that includes the forming ovipositor, which are not subject to the full force of the wasp innate immune system.

Study of diapause larvae indicate that *N. vitripennis* likely regulates *A. nasoniae* infection to prevent it from becoming pathogenic. We observed *N. vitripennis* infected with *An*‐GFP produce melanized nodules, a sign of an immune response to suppress *A. nasoniae*. In the majority of cases the host is able to successfully regulate *A. nasoniae*, but occasionally diapause larvae die with symbiont overproliferation observed. It is not known what causes this pathological phenotype, but it is tempting to speculate that these may represent male hosts that survived embryonic male‐killing. Our observations are consistent with data on *S. glossinidius* – tsetse interactions in which host‐immune activation is observed and controls symbiont titre (Weiss *et al*., 2008), but provides a contrast to symbioses of insects with *S. poulsonii* or *Wolbachia*, where host‐immune activation is not strongly induced by symbiont presence (Bourtzis *et al*., 2000; Hurst *et al*., 2003; Anbutsu and Fukatsu, 2010; Herren and Lemaitre, 2011). It is the first vertically transmitted symbiosis in which host nodulation responses have been observed, suggesting that the host/microbe interface is much more dynamic and ‘pathogen‐like’ than observed for other cases. This pattern is likely a consequence of active invasion processes through the gut, extracellular presence of the symbiont within the host, and the presence of intact cell‐wall structures typical of Gram‐negative bacteria. This biology is consistent with the genome of this heritable microbe, which encodes a diverse array of toxins and type III‐secreted effectors, and bears many of the hallmarks of a pathogen (Wilkes *et al*., 2010).

Finally, this study has demonstrated the tractability of the *N. vitripennis*/*A. nasoniae* system to genetic manipulation. Combined with the ease of culture of the wasp host both within fly pupae and in artificial media (Werren and Loehlin, 2009; Shropshire *et al*., 2016), the *An*‐GFP‐labelled stock enables in vivo real‐time tracking of symbiont invasion and persistence. The ease of establishing these patterns will enable study of mutants – both naturally occurring and engineered – on the symbiosis phenotype, and the pOM1 plasmid itself can be used to both complement mutations and perform gain‐of‐function assays. This toolkit will then establish the system as a model to understand the genetic basis of symbiosis between a gammaproteobacterium and an insect – that would itself represent a model for many such biologically important interactions.

## Experimental procedures

### Nasonia vitripennis, *symbiont strains and routine maintenance*

*Arsenophonus nasoniae* (strain Fin'13, isolated from Turku, Finland in 2013) was grown in BHI medium at 30°C and 250 r.p.m. for 6 days until an OD_600_ = 0.6–0.8 was obtained. *Nasonia vitripennis* strain AsymC was maintained using *Sarcophaga bullata* fly pupae as hosts. Five fly pupae (up to 30 days old) and five mated females were added to each tube. The tubes were sealed with cotton wool and placed in a 25°C incubator with 14:10 L:D cycle for 14–15 days until the new wasp generation merged.

### *Genetic manipulation*

*Arsenophonus nasoniae* was grown in 50 ml of BHI. After this period, the culture was spun down in 50 ml Greinier tubes at 20 000*g* for 10 min. The pellet was re‐suspended in 10% sterile glycerol and washed five times using 1 ml of sterile 10% glycerol in a regular Sigma 1–14 centrifuge at 16 160*g* for 1 min, and re‐suspended in 50 μl of the same solution. The pellet was then placed on ice for 10 min, 200 ng of plasmid pOM1‐*gfp* were added and gently mixed in the suspension. The mixture was then placed on ice for 10 additional minutes and electroporated in a 1 mm cuvette using 1 pulse at 2.6 kV in a micropulser electroporator (Bio‐Rad, UK). Immediately after, 1 ml of BHI was added to the cuvette and the suspension was placed in a regular 5 ml sterile vial and allowed to recover at 30°C and 250 r.p.m. over 24 h. After this period the culture was pelleted and plated in BHI agar plates containing 50 μg ml^−1^ spectinomycin (Sigma). The plate was sealed to avoid desiccation and placed at 30°C for 6 days until tiny colonies appeared on the agar surface. GFP fluorescence was detected in a M165 FC Leica stereoscope equipped with a Leica EL6000 external light source for fluorescence excitation and visualized using the GFP plus filter [480/40 nm (460–500 nm); Leica Microsystems (UK) limited] using 20×–60× magnification. A GFP‐positive colony was selected and grown in BHI + spectinomycin under the same conditions as the wildtype strain, labelled *An*‐GFP and stored at −80°C till further experiments.

### *Infecting* N. vitripennis *with* A. nasoniae

An efficient and fast infection protocol was developed to infect *N. vitripennis* with *A. nasoniae. Arsenophonus nasoniae* was grown in BHI, pelleted and re‐suspended in 10% sterile glycerol, as previously described, and used to inject 10 fresh *S. bullata* pupae. The pupae were surface sterilized with 70% ethanol to reduce potential contamination during the injection process. Bacterial inoculations were performed using 0.2 mm diameter needles and a small drop was injected in the junction between pupal segments. After this, the pupae were placed in a regular fly plastic vial where a tiny amount of absorbent paper had been placed at the bottom to avoid condensation and to dry the excess of liquid medium used in the injection. The use of absorbent tissue is important as the presence of moisture exponentially increases the chances of the fly pupae to get spoiled and/or the wasps to drown in liquid drops. Five to 10 min later, 10‐mated *N. vitripennis* female wasps were placed in the tube. Nine to 10 days after, the fly pupae were opened to collect the *N. vitripennis* pupae, which were examined under a M165 FC Leica stereoscope equipped with GFP epifluorescence. Non‐injected pupae were used as negative control to determine patterns of autofluorescence.

*An*‐GFP positive *N. vitripennis* pupae were placed in a separate tube and allowed to emerge from their pupal case after day 14. Wildtype *N. vitripennis* males were added as necessary to mate with the *An*‐GFP infected wasps. *Nasonia vitripennis* infected with *An*‐GFP were then maintained using standard maintenance outlined above, and monitored periodically for maintenance of the *An*‐GFP through examining fluorescence in *N. vitripennis* pupae.

### *Analysis of* A. nasoniae *transmission through* N. vitripennis *males*

Male killing by *A. nasoniae* is not complete, and we tested the capacity of infected *N. vitripennis* males to transmit *An*‐GFP. To this end, fly pupae parasitized with *N. vitripennis* carrying *An*‐GFP were cracked at the L3 stage (day 9–12) to ensure that all pupae were GFP positive and returned to the incubator till wasps emerge from the pupal case at day 14–15. *An*‐GFP males were collected. Virgin *N. vitripennis* females lacking *An*‐GFP were collected concurrently. Subsequently, one *N. vitripennis* male carrying *An*‐GFP was added to a vial containing five virgin females and after 24 h, five fly pupae were added to allow oviposition. This procedure was repeated with a total of 10 males and 50 females. Fly pupae were cracked 10 days after and the *N. vitripennis* pupae examined under the microscope for the presence of GFP. Lines were then maintained over three generations to establish if a signal of *An*‐GFP presence could be detected.

### *Visualizing* A. nasoniae *infection and localization during* N. vitripennis *development*

A total of 70 tubes containing five fly pupae and five mated *N. vitripennis* females from the stock carrying *An*‐GFP were established, alongside an equal number lacking *An*‐GFP as negative control. Females were allowed to oviposit for 24 h and afterwards removed to 25°C to maintain synchronized wasp embryos during the experiment. Each day, five vials of each treatment (*N. vitripennis* ± *An*‐GFP) were picked from the incubator. The fly pupae were cracked and the fly pupal surface and wasps therein were examined for the presence and localization of *An*‐GFP infection. The number of wasp larvae and pupae infected with *An*‐GFP was noted, and images captured for later examination.

### *Light‐sheet microscopy of* N. vitripennis *pupae carrying* A. nasoniae‐*GFP*

*Nasonia vitripennis* pupae carrying *An*‐GFP were collected at day 11 post‐oviposition. The presence of GFP was confirmed using a M165 FC Leica stereoscope equipped with GFP, and a single *An*‐GFP positive *N. vitripennis* pupa was prepared for Light‐Sheet microscopy. Briefly, 1% (wt/vol; Agarose low melt, Roth, Germany) was melted in a heat block at 82°C. The melted agarose was allowed to cool to ±50°C. A drop was poured on the wasp pupa and the mixture was sucked on a glass capillary tube (GMBH, Wertheim, Germany), allowed to solidify and visualized in a Z.1 Light‐Sheet microscope (Zeiss, Germany) with a 10×/0.2 illumination objective and a 20×/1.0 UV–VIS detection objective, using Zen software (Zeiss, Germany) for image acquisition and processing. Images were acquired using a pco.edge scientific complementary metal‐oxidase‐semiconductor (sCMOS) camera (PCO, Germany).

### *Visualizing* A. nasoniae *infection in diapausing* N. vitripennis

Fifty newly emerged and mated female *N. vitripennis* infected with *An*‐GFP and 50 wildtype females were placed individually in cotton‐plugged plastic vials with two host pupae. Each female was presented with two new host pupae every other day for 10 days in a 15°C incubator with 8:16 L:D cycle to induce diapause in the F1 larvae. Once the females had been moved onto new hosts, the parasitized pupae were moved to a 25°C incubator with 14:10 L:D cycle for 1 month. All parasitized pupae before day 10 were discarded and after 1 month the day 10 pupae were scored for diapause presence by opening the host pupae. The infection status of all broods was assessed.

## Supporting information


**Figure S1.** Colony of *A. nasoniae* carrying pOM1‐GFP (A, left) and wild type (B, right) under white light (top) and epifluorescence (bottom). Scale line = 1 mm.

**Figure S2.** In vitro growth of *A. nasoniae* WT (circles) and An‐GFP (triangles) at 30 °C and 180 rpm.

**Figure S3.** Development of *N. vitripennis* without An‐GFP infection, visualized under epifluorescence to estimate autofluorescent properties of *N. vitripennis* and the fly pupa. Scale bar = 5 mm.

**Figure S4.** Fraction of *N. vitripennis* larvae and pupae scoring positive for An‐GFP at different time periods following egg laying. Error bars represent 95% binomial confidence intervals.

**Figure S5.** The fraction of *N. vitripennis* larvae infected with An‐GFP that shows systemic infection (i.e. infection disseminated across tissues). Data given over time since oviposition. Error bars represent binomial confidence intervals.

**Figure S6.** Ovipositor and oviposition apparatus of Nasonia vitripennis. A) detached ovipositor from individual infected with An‐GFP B) detached ovipositor from uninfected female (autofluorescence control) C: detached ovipositor attached to fly pupa D: Magnified image of the oviposition apparatus, showing bright green fluorescence in the distal area. Scale bar (blue) = 0.5 mm.
