# Supplementary figures and images for: Genetic manipulation allows in vivo tracking of the life cycle of the son‐killer symbiont, Arsenophonus nasoniae, and reveals patterns of host invasion, tropism and pathology
Source: Environ Microbiol. 2019 Jul 11;21(8):3172–82. doi: 10.1111/1462-2920.14724 (PMC6771839; doi:10.1111/1462-2920.14724)

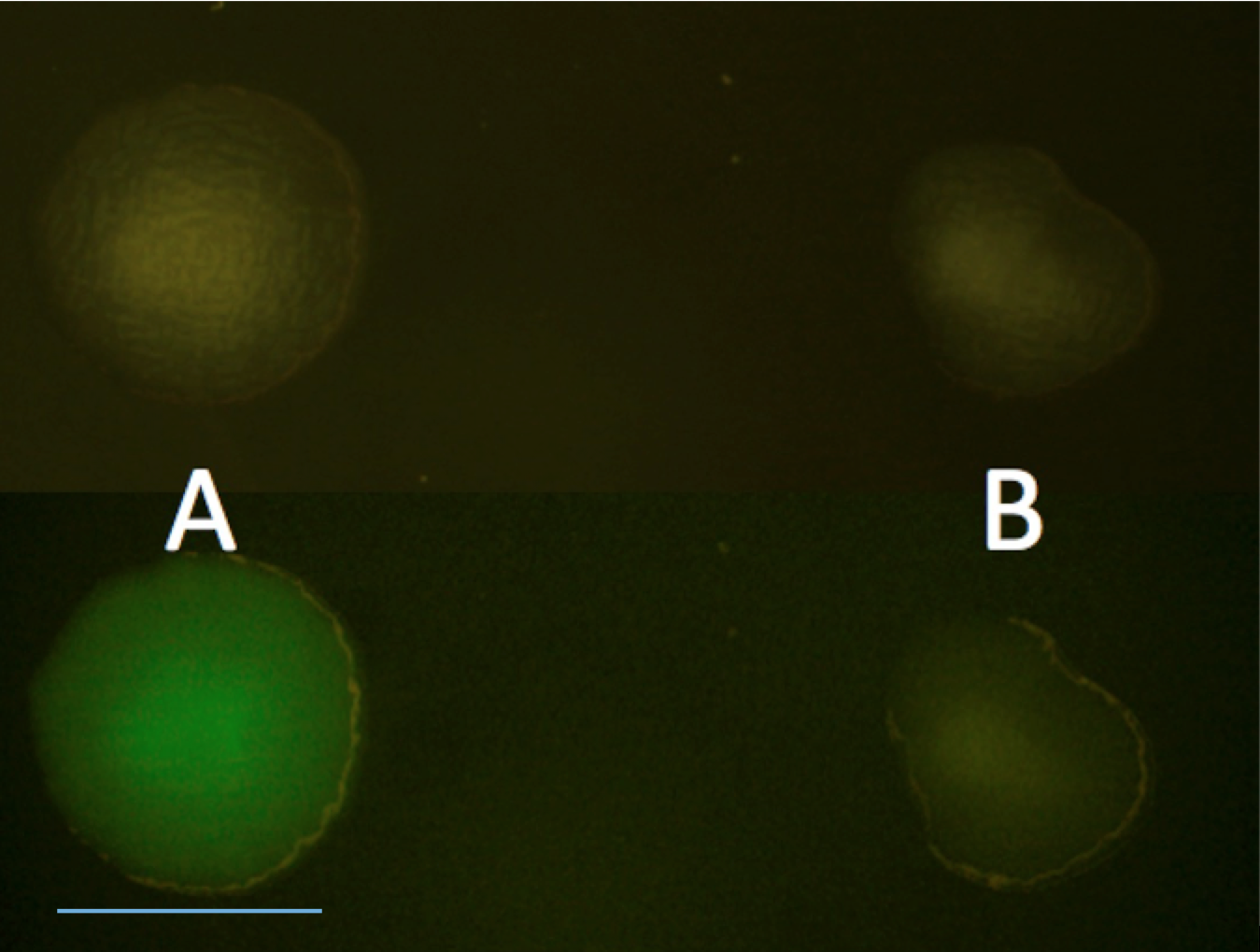

Supplement: Supplementary file 1 — Figure S1. Colony of A. nasoniae carrying pOM1‐GFP (A, left) and wild type (B, right) under white light (top) and epifluorescence (bottom). Scale line = 1 mm. [file EMI-21-3172-s001.tiff]

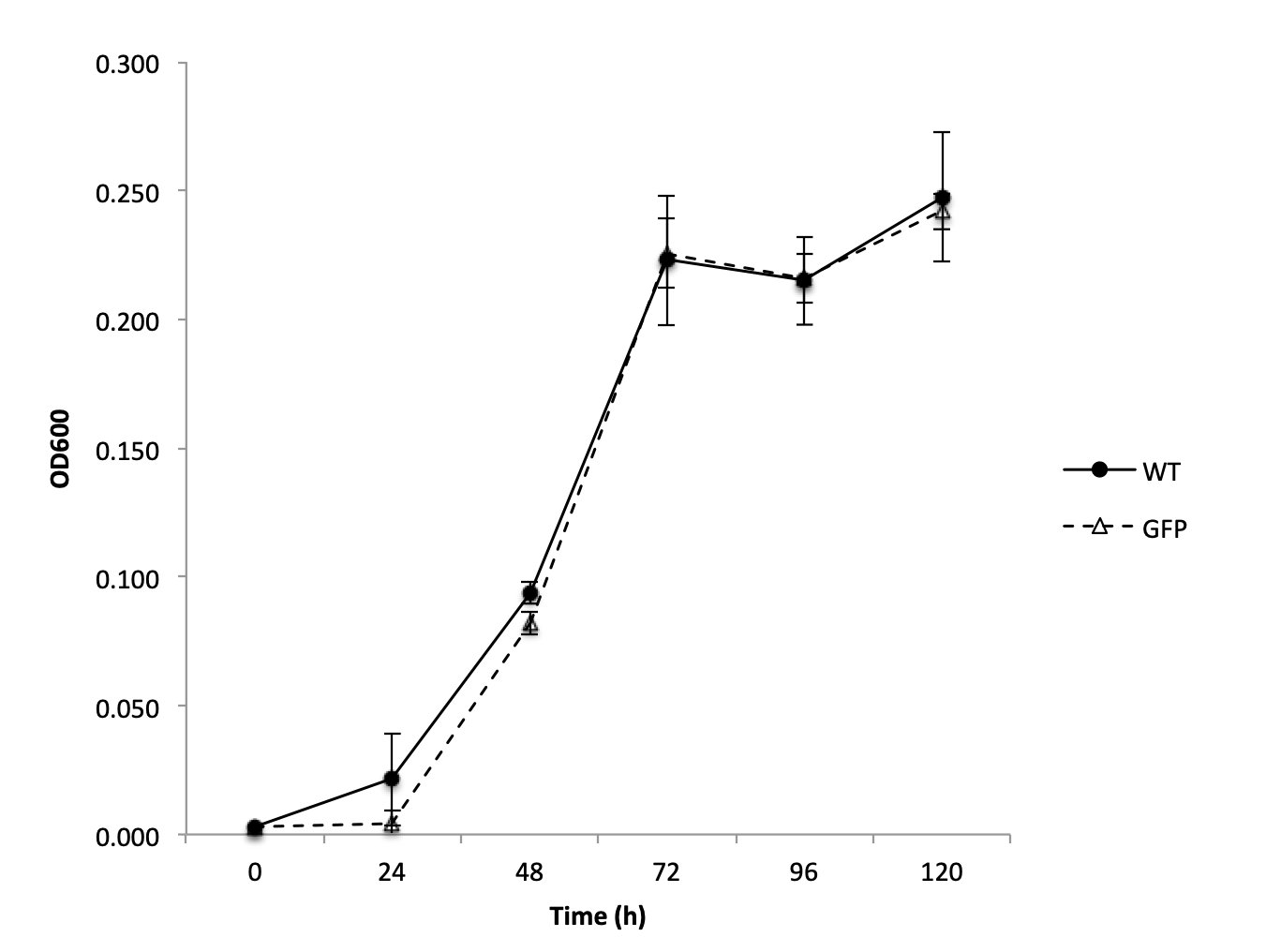

Supplement: Supplementary file 2 — Figure S2. In vitro growth of A. nasoniae WT (circles) and An‐GFP (triangles) at 30 °C and 180 rpm. [file EMI-21-3172-s002.tiff]

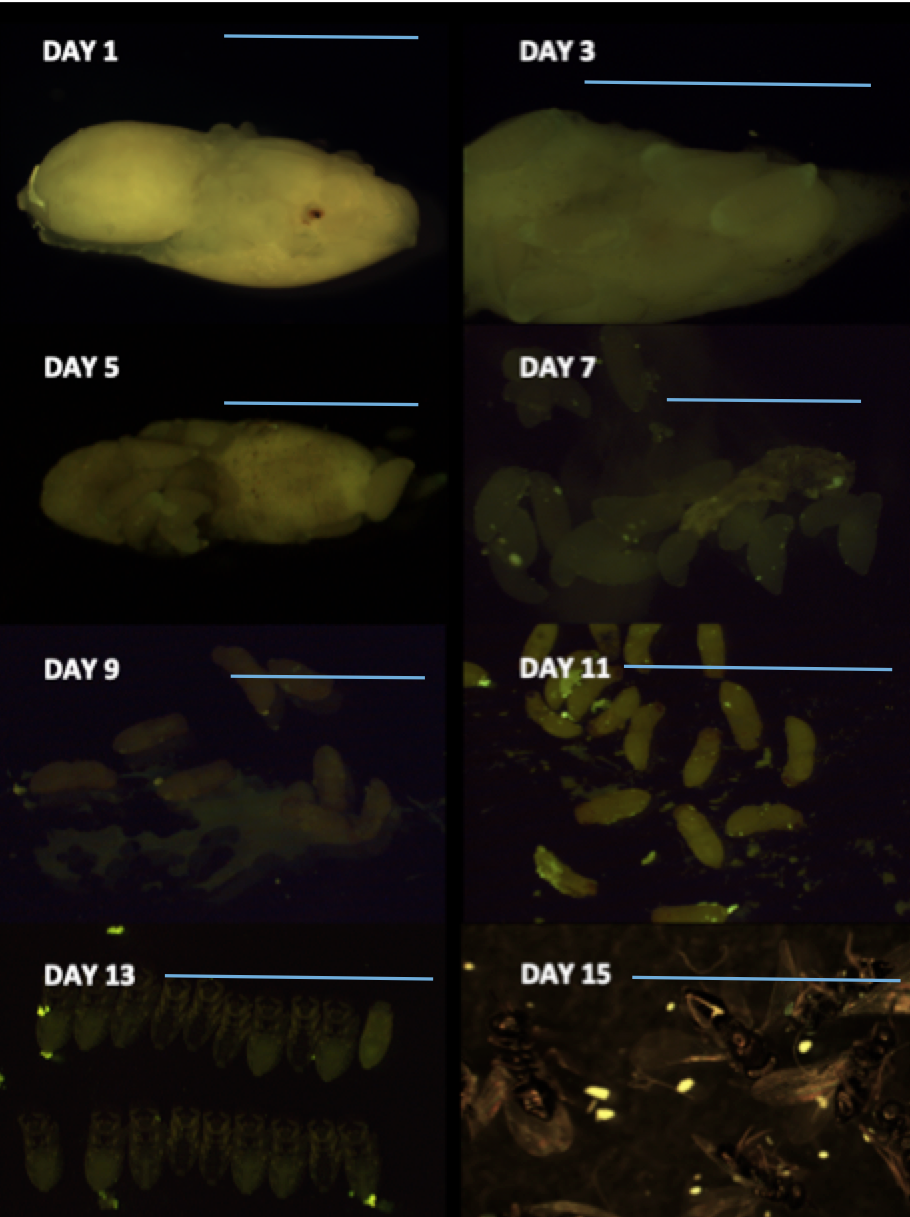

Supplement: Supplementary file 3 — Figure S3. Development of N. vitripennis without An‐GFP infection, visualized under epifluorescence to estimate autofluorescent properties of N. vitripennis and the fly pupa. Scale bar = 5 mm. [file EMI-21-3172-s003.tiff]

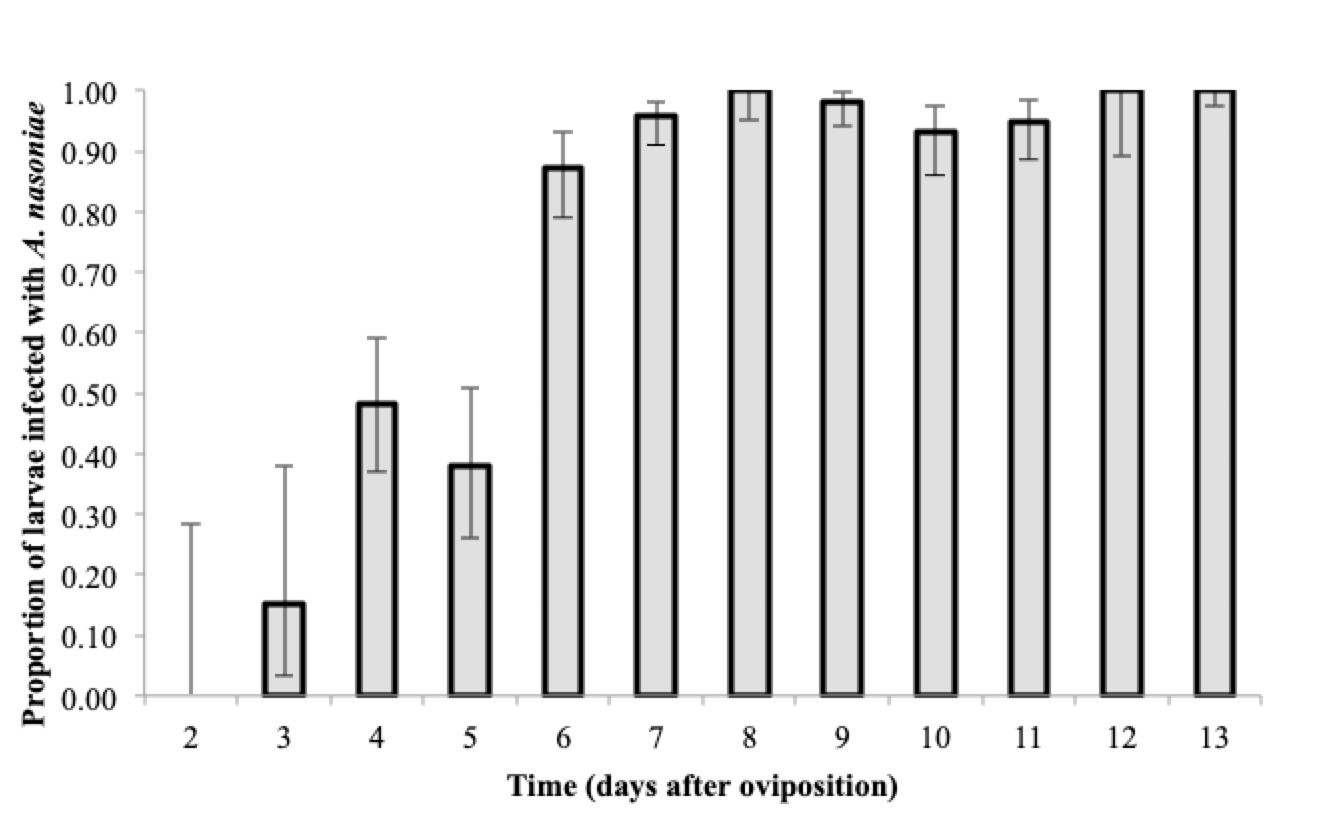

Supplement: Supplementary file 4 — Figure S4. Fraction of N. vitripennis larvae and pupae scoring positive for An‐GFP at different time periods following egg laying. Error bars represent 95% binomial confidence intervals. [file EMI-21-3172-s004.tiff]

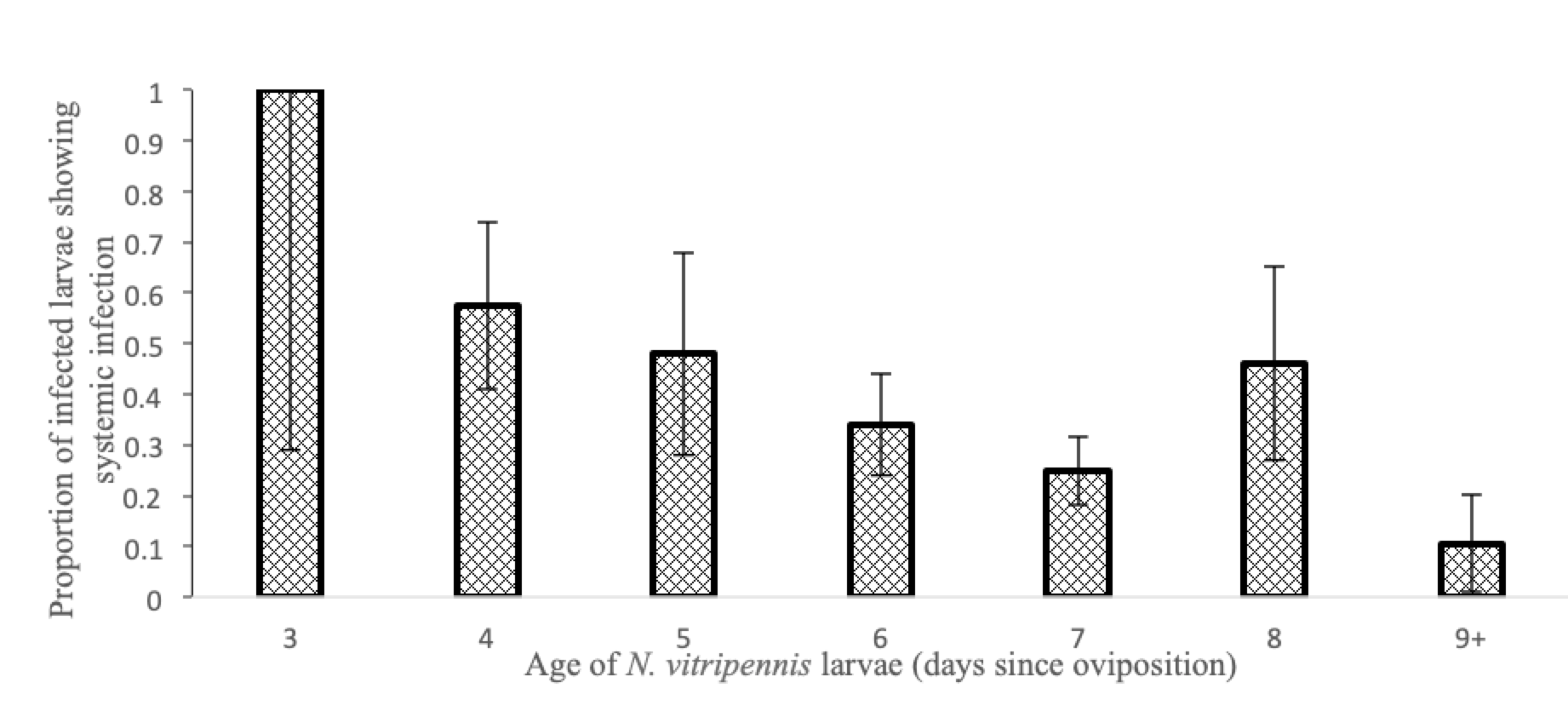

Supplement: Supplementary file 5 — Figure S5. The fraction of N. vitripennis larvae infected with An‐GFP that shows systemic infection (i.e. infection disseminated across tissues). Data given over time since oviposition. Error bars represent binomial confidence intervals. [file EMI-21-3172-s005.tiff]

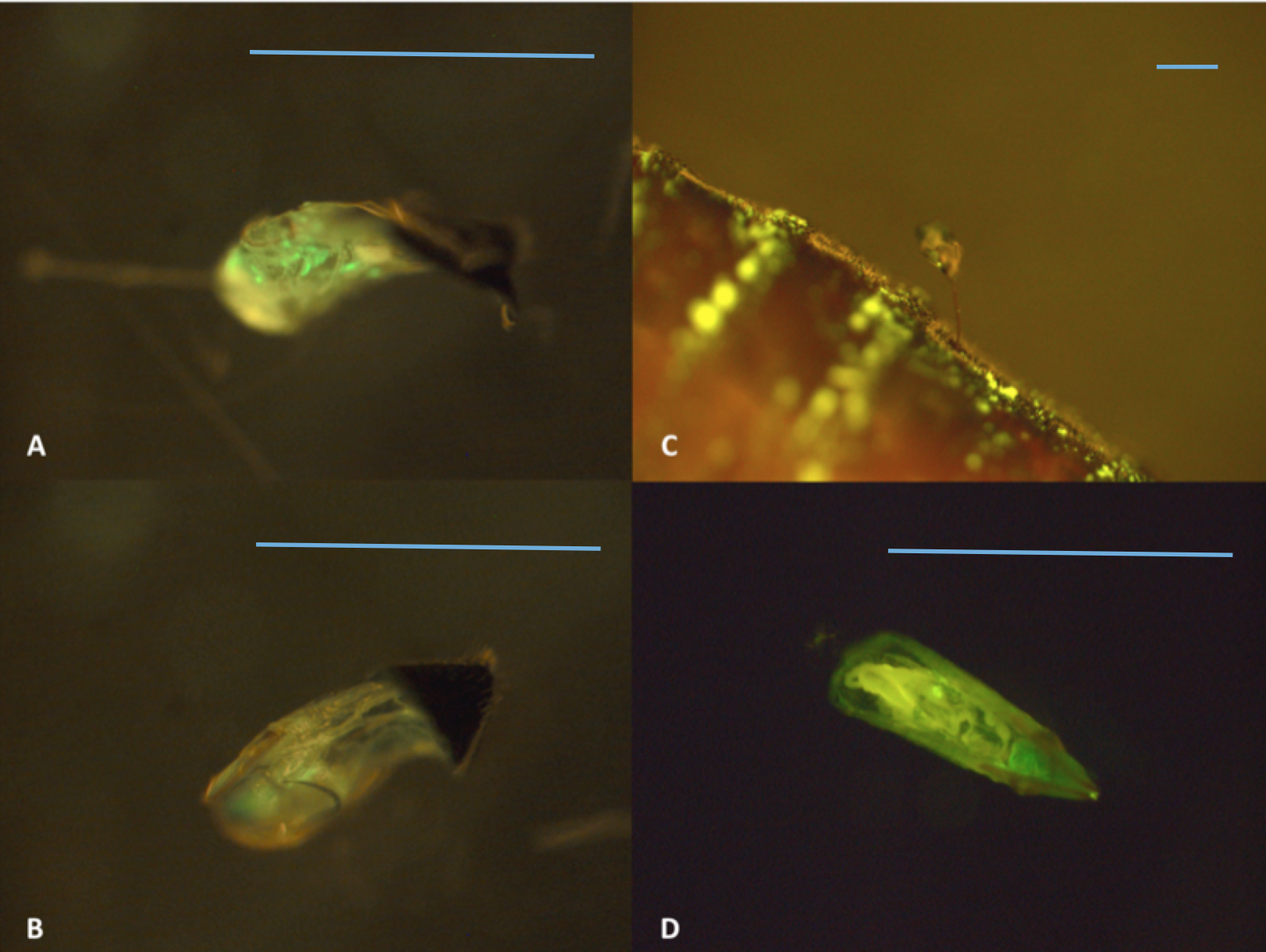

Supplement: Supplementary file 6 — Figure S6. Ovipositor and oviposition apparatus of Nasonia vitripennis. A) detached ovipositor from individual infected with An‐GFP B) detached ovipositor from uninfected female (autofluorescence control) C: detached ovipositor attached to fly pupa D: Magnified image of the oviposition apparatus, showing bright green fluorescence in the distal area. Scale bar (blue) = 0.5 mm. [file EMI-21-3172-s006.tiff]
